# Illinois State Board of Health

**Published:** 1881-11

**Authors:** 


### Article X.

### Illinois State Board of Health.

The regular quarterly meeting of the Illinois State Board of Health was held at the Grand Pacific Hotel, Chicago, Thursday and Friday, Sept. 29 and 30, 1881.

All the members were present, viz: T. M. Gregory, LL.D., President; Anson Clark, M.D.; W. A. Haskell, M.D.; R. Ludlam, M.D.; N. Bateman, LL.D.; John McLean, M.D.; John H. Rauch, M.D., Secretary.

The minutes of the previous meeting were read and approved.

Dr. Jas. M. Bryden, having been previously notified that complaints had been lodged against him for advertising improperly, and contrary to the rules of the Board, appeared in his own defense. He is a graduate of the College of Physicians and Surgeons, of Indiana; formerly practiced in the city of Chicago, but is now practicing in Fond du Lac, Wis., and has been advertising in the papers of that city, calling himself the *Scotch* physician, and "displaying" a wood cut of himself. He admitted that he left the State because he thought his certificate would be revoked if he continued his advertisements.

*Moved*, That on condition of withdrawal from the papers of all advertisements except the ordinary physician's card, and without the wood-cut of his portrait being inserted, the prosecution of this case of Dr. Bryden shall be dropped. Carried.

The following resolution, introduced by Dr. McLean, was adopted by the Board:

*Resolved*, That in all cases where information comes to the notice of the Secretary of the State Board of Health that persons holding certificates from said Board are advertising in an unprofessional manner, or in any way are guilty of unprofessional and dishonorable conduct, it shall be the duty of the Secretary to notify them at once to appear before the Board, and show cause why their certificates should not be revoked.

Dr. Ludlam offered the following notice to appear before the Illinois State Board of Health:

WHEREAS, Complaint has been made at this office that, as a licentiate of this Board, you have been guilty of irregular and unprofessional conduct; and whereas, if such charges should prove to be true, it will be necessary, under the statute, for the Board to withdraw your certificate, therefore you will please be notified herewith that an opportunity will be afforded you at the next meeting of the Board to present such papers and evidence in explanation and defense as you may have to offer in said case.

The case of Julius L. Lins, of Wallingford, Will county, was then considered. Lins made an affidavit in 1878 that he was a graduate of the University of Marburg, Germany, and had practiced medicine in Illinois ten years prior to July 1, 1877. Because of his ten-years practice he was not compelled to present his diploma for verification, *at that time.* The truth of this affidavit being questioned, the Dean of the Faculty of the University of Marburg was addressed, and the fact that he had never graduated there was ascertained. The Board ordered that his certificate be revoked and cancelled.

President Gregory laid before the Board a letter from Dr. G. H. Leach, of Cairo. The letter complained that W. H. Marean, of Cairo, had a certificate of practice from the Board to which he was not entitled. Intimations were also made as to improper methods of issuing certificates by the Board.

Dr. Rauch offered the following, which was adopted:

WHEREAS, Dr. G. N. Leach, of Cairo, in a communication to the Attorney-General of this State, dated Aug. 23, 1881, made certain charges in connection with the case of Dr. Marean, of Cairo; therefore, be it

*Ordered*, that Drs. Leach and Marean be cited to appear before this Board at its next meeting, to show cause why the certificates issued to each of them should not be revoked.

Dr. Rauch read his quarterly report as Secretary, of which the following is a brief synopsis:

Since the last meeting 553 letters and 421 postal cards have been written; 5,552 circulars and 1,850 packages have been distributed, including 1,820 reports of the Board and Register of Physicians and Midwives. Certificates have been issued to 92 physicians on diplomas, 5 on years' practice, and 3 upon examination; 8 to midwives on diplomas or certificates of examination, (nearly all from foreign institutions), 3 on years of practice, and 5 after examination. One diploma has been held for further consideration, one was withdrawn while being considered, and 23 doctors were compelled to leave the State because of their inability to comply with the law. An inquiry has been received from Portland, Oregon, where a diploma from the Edinburgh University, of Chicago, was presented to the Medical Director of the A. O. U. W. He was at once informed of the fraudulent character of that institution, and the action of the Board with regard to the same. The distribution of the official register makes the work of the Board less onerous, although there is still considerable annoyance from itinerant quacks who claim the benefit of the ten-year exemption clause.

In view of this fact, I would respectfully recommend that the Board take the necessary action, defining that section of the statute (*vide infra*).

Small-pox has appeared at five new points, and it will continue to occur while immigration is so heavy from Europe. Because of this probability, the Board should urge the protection of all by vaccination and re-vaccination, and I would also urge the Board to pass a rule making it obligatory upon the authorities of any township, town or city, to report promptly to this Board the outbreak of any contagious or epidemic disease, as I believe it would be of the greatest benefit to all parties concerned. The experience of the last nine months, especially in connection with smallpox, has impressed me with the necessity of the preparation by the Board of a sanitary code of ordinances, for the adoption by local authorities throughout the State (*vide infra*).

Another important matter to which my attention has been frequently called during the last three months, is the condition of the Illinois River. In view of the sanitary importance of improving its polluted condition, it is clearly the duty of the Board to do all it can to hasten the construction of the pumping works at Bridgeport.

RAILROAD SANITATION.

Although a subject of the utmost importance, and one affecting a large majority of our population, it has not yet received the attention which it demands. The hygiene of the emigrant trains is notoriously bad, but even the more elegant means of travel are far from faultless. During the past year travel on all lines has been exceedingly heavy, and this has tended largely to aggravate the already existing evils.

As bearing directly on this subject, I call the attention of the Board to the following correspondence:

OFFICE OF THE SECRETARY,
SANITARY COUNCIL OF MISSISSIPPI VALLEY,
CHICAGO, May 24, 1879.

*To* COL. D. N. WELCH, *General Superintendent Pullman Palace Car Co.:*

SIR: Enclosed herewith I transmit, for your consideration, a slip containing the substance of a discussion on railway sanitation, before the National Board of Health, on May 7, at Atlanta, Georgia, as also the propositions adopted by the Sanitary Council of Mississippi Valley.

Will you please inform me what measures are now adopted by your company to keep the sleeping-cars in good sanitary condition; and also what you did last summer and autumn, during the prevalence of yellow fever, and what disposition was made of the cars in which yellow fever patients were carried?

In this connection I beg to offer certain suggestions, upon which I should be pleased to have an expression of your judgment. In my opinion, the upholstering of parlor and sleeping-cars is too heavy, either for comfort or health, in summer. Could they not be put in what might be called a *summer dress* from May to September, the seats be furnished with cane bottoms and backs, or covered with leather or enamelled cloth? or, if the plush upholstery must be retained, then cover it with linen or other material that could be often changed and washed; substitute the same material for the heavy woolen curtains, and dispense with carpets entirely? Woolen stuffs absorb and retain waste matters much more readily than linen, and are much more difficult to cleanse; consequently they are much more liable to convey infection. From a strictly sanitary standpoint, it would probably be better to construct a different style of sleeping-car, for use exclusively south of latitude 37° N. A car starting from Chicago and running through to New

Orleans is frequently subjected to great extremes of temperature. Unless provision can be made both for summer and winter ventilation, such extremes must necessarily be deleterious to health, aside from the question of comfort.

I am also of opinion, in common with other sanitarians, that long distance through-cars—as, for example, from this city to New Orleans, or to New Yoik, or from New Orleans to Washington, are not conducive to public health, but, on the contrary, are often promoters of contagious and infectious diseases. The change to a fresh and clean car at Cairo, Pittsburgh, Buffalo, or Atlanta, as the case might be, would no doubt diminish the risk of contagion or infection, if, indeed, it did not add to the safety of the traveling public in other ways; and such a change could be made with little inconvenience or trouble to passengers.

Very respectfully,

JOHN H. RAUCH, *Secretary.*

PULLMAN PALACE CAR COMPANY,
GENERAL SUPERINTENDENT'S OFFICE,
CHICAGO, June 16, 1879.

JOHN H. RAUCH, M.D., *Secretary Sanitary Council of Mississippi Valley, Chicago.*

DEAR SIR: In reply to your letter of the 24th ult., asking for information as to what measures are now adopted to keep sleeping cars in a good sanitary condition; also what we did last summer and autumn, during the prevalence of yellow fever, etc., I have to say that, immediately on receipt of your letter, I forwarded it to the General Superintendent of our Pullman Southern Car Company. After receiving his full report on the subject, I forwarded the papers to the Superintendent Central Division of the Pullman Palace Car Company, whose reports, and also that of Major Hays, General Superintendent P. S. Car Company, will be found herewith.

You will perceive, by examination of the two reports, that the questions you ask are pretty thoroughly answered therein; therefore it is not necessary for me to go particularly into details. Ever since the organization of the Pullman Palace Car Company, up to the present time, the question of the free use of disinfectants and detergents has been one to which the company has given much attention. The formula for the disinfectant now in use is the one recommended by the American Public Health Association, at a session held in New York in 1872 or 1873, and since its adoption it has been constantly in use in our cars; and we are informed by medical men and chemists that there is no better disinfectant for the purposes for which it is required. We had passed through two seasons of yellow fever on our coast lines before the extraordinary epidemic of last year, so that we were pretty well prepared to meet the emergency.

Whenever we have had a case of fever, the car has been thoroughly fumigated with sulphur, then laid by until it was believed that the germs of contagion were entirely destroyed. We are very certain that the disinfectants are thoroughly used in the cars, as the matter is continually being reported upon by our inspectors and special service men, and we are equally sure that our linen is clean on every trip, that the carpets are taken up and well beaten, and the plush and other interior arrangements of the cars thoroughly cleansed. The mattresses are frequently re-upholstered, and the pillows, blankets, etc., are thoroughly cleaned at stated times, and if inspection shows that anything of that nature requires treating oftener, the inspector immediately makes a report to that effect to this office.

In regard to the matter of ventilation, we will be very glad to receive any suggestions that any Sanitary Council may see fit to make. We are constantly experimenting with new ventilators. Most of the so-called inventions, however, are impracticable. Such as we find to be of benefit, we adopt. We are now experimenting with several styles of ventilators, one owhich, at least, we hope will be found of some benefit.

In regard to making short runs, we have no doubt that in a sanitary point of view short runs would be better for the passengers, but that is a matter we cannot control. The railroad companies and the traveling public are in favor of long lines. Passengers will submit to a great deal of discomfort rather than change cars, and it is probable that it will be some time before their views will be altered.

Regarding your suggestion that cars should be built for a climate south of lat. 37°, which should consist of entirely different interior fittings to those used in the north, I would say that such a change would entail upon the Southern company a great expense. We made the attempt several years ago to furnish sleepers to a Southern road with hard finished floors without carpets, but on arrival of the cars at their destination, the railroad Superintendent ordered carpets put down at once, as he was afraid his passengers would think they were being provided with second class accommodations.

At the same time, if we had cars fitted solely for southern summer travel, we should be obliged to lay them up in winter, and provide others for northern people making winter trips south. At the present time, with limited travel, the carrying out of your suggestion would be too expensive for us. We would be very glad, for such cars as are used on the southern roads to supply hard wooden floors to them as fast as they go into shop, and not use carpets in summer. This is a matter that we will confer with the southern Superintendent about as early as practicable.

In conclusion, I would say that we will be very happy to hear any suggestions from the Sanitary Council in regard to matters discussed in their convention at Atlanta in which we are interested, and so far as practicable will be pleased to adopt them.

Very respectfully,

D. N. WELCH,

*Gen. Sup't P. P. Car Co. and Vice Pres. P. S. Car*

PULLMAN SOUTHERN CAR COMPANY,
GENERAL SUPERINTENDENT'S OFFICE,
LOUISVILLE, May 29, 1879.

*Respectfully returned to Col. D. N. Welch, Vice-President, Chicago.*

I have carefully read the enclosed clipping, from the Atlanta *Constitution*, of the proceedings of the National Board of Health, relating to the yellow fever epidemic of 1878; Dr. Rauch's letter and your endorsement of the 27th inst., referring the papers to me for a report of the measures used by the company to prevent the spread of the disease by means of our cars, etc.

In reply, I would state that early in July of 1878, when the Vice-President and myself were in New Orleans, there were rumors of some few cases of yellow fever, and not a few predictions of an epidemic; and at this early date, to guard against probable emergencies, additional sanitary measures were inaugurated, and the use of disinfectants in our cars was commenced. We sought and obtained the best medical advice as to the use of disinfectants, and before the disease had developed itself we were using every precaution, under a well-defined system; and by the time the tide of travel had set northward, this system was in full operation under rigid discipline.

All our cars were thoroughly disinfected at *every terminus of our lines*, and pure carbolic acid was exposed in open vessels in every car while *en route.* At terminal points every car was thoroughly cleaned—all bedding, seats, carpets, and in fact everything movable in the car, were taken out, whipped, brushed, and then fumigated in a close room with sulphur. Each car was scrubbed inside and out, and then closed and fumigated with sulphur. After this process with car and equipments, they were both exposed to the open air for several hours, carefully dusted, and both car and equipments liberally sprinkled with carbolic acid. Carbolic acid was put in each spittoon on every car running into or through any infected district five or six times during each day or night. All cars were thoroughly ventilated while *en route*, by open doors, windows and deck sash.

As soon as the disease was declared epidemic in New Orleans, Memphis and other points, our cars were withdrawn, as in the case of the lines from New Orleans to Chicago and St. Louis, from Louisville to Memphis and Little Rock, and from Cincinnati to New Orleans, via Montgomery. We had one line of sleepers only that continued unbroken during the entire epidemic—the line from New Orleans to Cincinnati, via Milan. On this line all cars were treated at each terminal point and *en route* as above indicated.

During the entire course of the epidemic we did not have a single case reported to us of yellow fever occurring from any *supposed infection* from our cars; and in *every instance* when any person coming from any infected district was taken sick on one of our cars, *not only the person*, but the *mattress, pillows, blankets and sheets as well, were removed at the first station, and all bedding and material thus removed was invariably destroyed—in no instance was any portion of it ever returned to a car.*

We do not know of a single case, *nor do we believe one can be found*, of yellow fever developing itself on any of our cars, *except in persons coming north from infected districts*, and in whose system, beyond a doubt, the germs of the disease were implanted before they undertook the journey. In every case of this kind (and they were very few), the persons, and everything movable in the car with which they had come in contact, were removed from the car as above described.

As before stated, all of our lines, except the *one* noted, terminating south in infected localities, were either contracted to the northern confines of the district in which the disease had been declared epidemic, or entirely withdrawn.

The one exception, from New Orleans to Cincinnati, via Milan, our cars were cleaned, and all the bedding and equipments therein handled at Cincinnati by men living in that city; and not only no case of yellow fever, but no case of disease of any nature, developed itself among any of these men during the entire summer and fall.

In contracting our lines as the disease manifested itself in Memphis and other points south, in the latter part of July and the early part of August all lines whose northern termini were in this city (Louisville) were entirely withdrawn, and thenceforward, until after the Board of Health had declared it safe for citizens to return to Memphis, *not a single sleeping car coming from an infected district came into this city;* the cars on the line from New Orleans to Cincinnati passed around the city by the short line cut-off, leaving the main stem of the Louisville and Nashville railroad three miles south of the city.

In conclusion, I would say, were steamboats as careful of their equipments and general sanitary condition—*were the traveling public one-half as careful of their clothing and baggage*, to prevent the spread of infectious diseases—as our service requires, I opine there would be less need of such strict quarantine measures ; but under the existing regulations, I am in favor of the most thorough investigations, in cases of epidemic, of all persons and things leaving any infected locality. We have always done everything possible to prevent the spread of disease by means of our cars—have done much more than the National Board of Health appears to require; and we would like to see such measures inaugurated as would prevent the transportation of infected clothing, merchandise, etc., as this is undoubtedly the source from which the most danger is to be apprehended.

THOMAS S. HAYS,
*General Superintendent.*

PULLMAN PALACE CAR CO., CENTRAL DIVISION,
SUPERINTENDENT'S OFFICE,
PHILADELPHIA, June 7, 1879.

Respectfully returned to Col. D. N. Welch, General Superintendent, having noted carefully the within correspondence, and give below a report of the manner of handling cars, bedding, equipments, etc., in Central Division.

All mattresses, pillows, seats and blankets are thoroughly aired, beaten or shook at the termination of each trip, which will make an average of three times per week that they receive this treatment.

As a disinfectant we use a mixture of carbolic acid and copperas, or carbolic acid alone, applying the same only in urinals and closets.

The only case of yellow fever that we had in any car of this division was in sleeping car No. 287 *en route*, St. Louis to Jersey City, September 13–15. A lady passenger, who entered the car at St. Louis, complained of feeling sick, but supposed it to be a case of chills and fever, until the train had arrived at or passed Pittsburgh, when the passengers in the car concluded it was a case of yellow fever. The car went through to its destination, Jersey City, and was immediately switched over into the side or freight yard, the authorities notified, a tug chartered, and the lady and her husband taken down to the quarantine station in New York harbor, some time about three or four o'clock in the morning.

The physician of the Penn. R. R. Co. was given charge of the car, and he immediately disinfected it, which he did in a most thorough manner, as our reports will show, the equipments and all the interior of the car, so far as metal work, etc., were concerned, being almost completely ruined by the use of acids. After they were through with disinfecting the car, it was sent to Altoona and remained in an exposed position during the winter, at a temperature of weather sometimes as low as 13° below zero. The car was then shopped for thorough and general repairs, costing nearly four thousand dollars.

As has been our custom, which we are still pursuing, all cars, at the termination of each and every trip, are thoroughly washed and cleaned, bedding and equipments aired and dusted, carpets taken up and beaten, and as you will see by your pay-rolls, we have quite a small army of cleaners doing this class of work.

E. H. GOODMAN,
*Superintendent.*

Since the foregoing correspondence I have traveled at least 45,000 miles in sleeping cars. I have paid especial attention to this subject, and have reached the conclusions which follow. The condition of the car depends on (a) the handling of the car by the conductor and porter; (b) the number of passengers; (c) the length of the run; (d) the season of the year.

It is but just to state that the sanitary conditions of the Pullman cars are better than any other cars, and from correspondence and personal interviews I am satisfied that the company would cheerfully adopt the suggestions, if the public demands the same. My excuse for calling attention to the subject at this time is to educate the public to the dangers incident to traveling in through cars.

In 1879 when no cars were allowed to cross the Ohio at Cairo, transfers were daily made, and not only was there no complaint but much commendation of the method. I would respectfully suggest that this Board take such action as may be deemed proper to the subject, feeling confident that the traveling public will cheerfully comply when the benefits are made known and the companies will gladly make the change.

DANGERS OF SUMMER RESORTS.

I was at Beach Haven, on the New Jersey coast, when the Parry House was destroyed by fire. The fire was discovered at 3 A. M. and as the building was constructed of the lightest material, it was completely destroyed within an hour.

It seems almost miraculous with the limited means of egress that no lives were lost. I have been in burning hotels, but never were the inmates so completely at the mercy of the flames as in this instance. As it was, several have since died from the exposure and excitement incident to the fire. This hotel is a type of the scores to be found on the New Jersey coast, and unless steps are taken to require adequate means of escape and facilities for putting out fires, it is only a question of time when great loss of life will occur. In view of the fact that during the season, thousands from other States are guests at these houses, I think it would not be out of place to suggest that our Board call the attention of the New Jersey State Board of Health and the Boards of Health in other States where such resorts exist, to the subject and to respectfully recommend that in case there are no laws concerning it, they make an inspection of these buildings covering these points, as well as the sanitary conditions that obtain in and around these houses. The publication of such a report would go far towards remedying the evil.

The report was discussed, and the following resolutions and orders therein suggested were duly adopted by vote:

WHEREAS, In several instances recently, when suits have been brought against parties for the violation of the "Act to regulate the practice of Medicine," advantage has been taken of the ten-year clause by those who do not strictly come under its provisions;" therefore,

*Resolved*, That it is decided by this Board that the ten-year exemption clause, as a qualification, means that the party who avails himself of the said exemption must have actually been engaged in the practice of medicine as a means of gaining a livelihood, and must have publicly professed himself to be a physician for a period of ten or more years before July 1, 1877; further,

*Resolved*, That parties who have only occasionally prescribed during that time, are not by this Board considered as entitled to the benefits of the exemption.

ORDER.

Under the authority conferred upon the State Board of Health by section 2, of the State Board of Health Act, it is ordered—that on and after Jan. 1, 1882, the first cases of small-pox occurring in any township, town or city, in this State, shall be promptly reported to the Board, and also the prevalence of any epidemic, by the local health authorities, it being borne in mind that in counties where township organization exists, the township board is the Board of Health, and in counties not under such organization, the county commissioners act in like capacity.

*Resolved*, That the Secretary of the Board be, and is hereby instructed to request the medical schools of the State to give one or more lectures each year on the relations of the profession to the public and to each other, believing the same to be in the interests of the people and of medical men.

Drs. Clark, McLean and Bateman, Committee on Railway Sanitation, reported as follows:

WHEREAS, Earnestly believing that many sanitary evils exist with regard to the management of emigrant trains, ordinary passenger trains, sleeping coaches, the grounds and buildings of depots and even the trains for the transportation of goods and merchandise, therefore,

*Resolved*, That the Secretary of this Board be instructed to communicate with the managing authorities of the various lines in this State, calling their attention to the matter and requesting them to take such steps as they consistently can with reference to the sanitary improvements in these respects.

John I. Spence, of Liberty, Adams county, having applied for a certificate of graduation, presented the diploma of the College of Physicians and Surgeons of Joplin, Mo, Upon motion of Dr. Clark, the following order was adopted:

WHEREAS, It appearing from the published announcement of Joplin College of Physicians and Surgeons at Joplin, Mo., that the said medical college is not conducted according to the custom of such institutions considered in "good standing," in that it recognizes three years of practice as equivalent to one course of lectures, and that the general character of the announcement is such as to make it evident that it is not conducted in accordance with the practice of medical schools that are considered in "good standing" by this Board; therefore,

*Resolved*, That this Board declines to issue a certificate to its graduates.

Three other practitioners actions were taken under advisement by the Board, and in one case discretionary power was given to the Secretary to revoke the license.

Dr. Bateman moved, that the Secretary be authorized and instructed by this Board to prepare and issue as soon as practicable, the necessary blanks, forms, circulars and instructions for the collection and tabulation of returns of vital statistics.

Upon motion of Dr. McLean, the Secretary was instructed to issue a circular calling the attention of college authorities to the requirements of this Board in respect to the good standing of Medical Colleges as published in its report.

The case of Dr. M. H. Pasco, of Chicago, was referred to the Secretary with power to withdraw the Board's certificate at his discretion, and report his action at the next meeting.

Drs. Rauch, Gregory and McLean were appointed a committee to report to the Board at its meeting as to what action, if any, was necessary on the subject of Maritime Quarantine.

The Board then adjourned.

